# Pregnancy, childbirth and postpartum experience in pregnant women infected with SARS-CoV-2 in 2020 in Paris: a qualitative phenomenological study

**DOI:** 10.1186/s12884-023-05406-x

**Published:** 2023-01-31

**Authors:** Jean-Sébastien Cadwallader, Laura Berlingo, Valentine Rémy, Marc Dommergues, Julie Gilles de la Londe

**Affiliations:** 1 Department of General Practice, Sorbonne University, 27 Rue Chaligny, Paris, 75012 France; 2 Sorbonne University, INSERM, Institut Pierre Louis d’Épidémiologie Et de Santé Publique (IPLESP), Paris, F75012 France; 3 Department of Gynaecology Ans Obstetrics, Pitié Salpêtrière Hospital, Assistance Publique - Hôpitaux de Paris (AP-HP), DMU Origyne, Sorbonne University, Paris, 75013 France; 4 Department of General Practice, Université de Paris, Paris, F-75018 France

**Keywords:** Qualitative research, Pregnant women, COVID-19, Phenomenological

## Abstract

**Background:**

The COVID-19 pandemic and the resulting lockdowns triggered social discontent on an unprecedented scale. Descriptive phenomenological studies showed that pregnant women were under intense stress during the COVID-19 outbreak, even though they remained uninfected. The purpose of this study was to report on the experiences of pregnant women affected by mild COVID-19 during the first wave of the pandemic.

**Methods:**

In this non- interventional qualitative study, we analyzed pregnant women’s experiences using an interpretive phenomenological analysis approach. We conducted semi-structured interviews with women who had had a mild COVID-19 during their pregnancy, and gave birth or planned to give birth in the maternity units of Sorbonne University in Paris, France.

**Results:**

Participants reported that at the time they had COVID-19, they were not afraid of being seriously ill, but of transmitting COVID-19 to their close relatives. Their main concern was being pregnant and becoming a parent in a world where the pandemic deeply altered social environment. This included uncertainty about the future and an acute feeling of isolation related to lockdown. The idea that their partner might not be allowed to attend childbirth was almost unanimously felt as intolerable. In contrast, women had positive feelings regarding the fact that lockdown resulted in a de facto paternity leave leading to a certain degree of equality in the couple regarding baby care and household chores. Unexpectedly, the pandemic social distancing measures helped participants escaping from behavioral constraints, including the unspoken rule that they should welcome greetings from friends and family, despite being exhausted by the recent birth.

**Conclusions:**

Our results suggest that avoiding separation from their partner is a key to benevolent medical care for pregnant women in times of health crises. The unexpected benefits women reported in a world of lockdown cast a new light on their expectation regarding parenthood today.

## Background

The COVID-19 pandemic and the resulting social and health crisis have triggered many scientific, epidemiological and sociological studies across the world. As early as May 2020, researchers warned internationally against the risk of psychological repercussions of the health crisis on pregnant women [1]. This was alarming, since pregnancy and parenthood, usually a source of joy, may trigger negative emotions related to birth, mental load, work and family at large [2–4].

In France, a lockdown took place between March 2020 and June 2020 with the emergence of the variant alpha. This lockdown consisted in the closing of artistic, commercial sectors considered as “non-essential” [5].

Some French studies described low mental well-being scores during this phase, especially in poor areas of the main cities such as Paris [6]. American studies showed that pregnant women who had not been infected were under intense stress during the COVID-19 outbreak with qualitative studies on going to understand the impact [7, 8]. No qualitative study has been carried out in France about pregnant women infected with COVID-19.

Our objective was to describe the lived experience of women diagnosed with a mild to moderate SARS-CoV-2 infection during pregnancy regarding pregnancy, childbirth, and early parenthood.

## Methods

We carried out a qualitative study using an interpretative phenomenological analysis (IPA) [9], corresponding to an in-depth study based on individual participants’ narratives about their experience of a phenomenon [10]. It is an inductive approach, not used to confirm pre-established hypotheses but to discover new concepts on the issue under study.

### Sample and recruitment

To achieve a homogenous sample, we took advantage of a list of pregnant persons infected with COVID-19 during their second or third trimester, who gave birth or planned to give birth in the maternity units of the Sorbonne University group (Pitié-Salpêtrière, Tenon and Trousseau Hospitals). The diagnosis of COVID-19 for our study was based either on a positive nasopharyngeal SARS CoV 2 PCR, or on highly suggestive clinical symptoms.

We contacted eligible persons by e-mail, following the chronological order of the list. The e-mail inviting them to take part in the study gave practical information together with a consent form. All the women who responded positively were interviewed.

The women interviewed were to have sufficient mastery of the French language, to be able to undergo a long interview, and to give verbal consent to participate in the study, having read the information sheet.

We discontinued recruitment when sufficiency of data and concepts was obtained [11].

### Interviews

Four researchers carried out the interviews: a senior researcher (LB), a methodologist specialized in qualitative research (JG), a psychologist (AA) and a junior researcher (VR).

The interviews were carried out at the participants’ convenience and with their consent, using digital tools (Whatsapp®, Skype®, Zoom®). Due to partial lockdowns during this period in France, it was very difficult to carry out face to face interviews. We chose to conduct online interviews. This simplified the recruitment of post partum women often at home with their children. A recent paper showed that online qualitative research is as efficient as face to face interviews [12]. All interviews were recorded, with the women’s consent. The final product of the transcription, the “verbatim”, was used as a basis for the analysis. All interviews were made anonymous, with ID codes generated on a secured computer. Interview transcripts were safely stored to protect confidentiality on a unique computer protected with a password. The interviewer remained as unobtrusive as possible to limit any influence on the interviewee. The interview guide was open, and the subjects to be discussed were suggested but not compulsory. Each interview was subjected to a debriefing session involving the four interviewers. This enabled the researchers to initiate the analytical process and to comment on the quality of the interviews in order to improve the interviewers’ skills.

### Analysis of the results

We used interpretative phenomenological analysis (IPA), a qualitative method developed to describe the “experience of illness”, particularly in the medical and psychological fields [13]. It is part of a dual hermeneutic perspective: the researcher gives meaning to the meaning attributed by the subjects to their own experiences. Every sequence of verbatim was analysed independently, with the emergence of codes for each interview, gathered in subthemes and categories in a second step, ending by the building of superordinate themes (the first step also named « coding»). The analysis was triangulated confronting the points of view of four researchers for each step of the analysis, with dedicated sessions every three interviews. Before these sessions, VR and JG coded independently every transcript. LB, VR, JG and JSC discussed during these sessions the codes for each interview and then later discussed for each interview the emergence of several themes. These themes were elaborated considering the common experience in the codes. At the end of the process, the superordinate themes were elaborated using all the themes emerged form all the interviews. Sufficiency as IPA required was obtained when new categories emerging from new interviews were similar to the previous ones, without new relevant information [11]. We did not use any software due to the few interviews required in the IPA method [9].

### Ethical aspects

The research protocol received approval from the Commission Nationale Informatique et Libertés (CNIL, Reference 2,218,112 v 0) and from the Research Ethics Committee (IDF Ile de France 4) (approval issued on June 25^th^, 2020 under n°2020-A01184-35).

## Results

The first 50 pregnant women in the “COVID-19 patients” list were contacted. Twelve responded and were interviewed from June to August 2020. None of them had to be excluded because of an insufficient proficiency in French. The material studied amounted in all to twelve hours and forty-five minutes, with a mean time of sixty-two minutes per interview. The women were aged 27 to 42. Nine of them had already given birth to their babies at the time of the interview (Table 1). All women partners were identified as men.

**Table 1:** Participants

P	Age Family situation before this pregnancy Type of household	Timing of the interview	Symptoms and PCR tests
P1	38 years old Single, no children Lives alone, duplex apartment, with no outside premises	Pregnant	Cough, fever, anosmia, ageusia, substantial dyspnoea PCR + 20WA
P2	27 years old Married, no children Lives in an apartment, with no outside premises	Pregnant	Anosmia, ageusia PCR + 25 WA
P3	34 years old With a partner, 2 children Accommodated in a social hostel	3 weeks post partum	Asthenia, myalgia PCR + 31 WA
P4	32 years old, Married, 1 child	3 weeks post partum	Pseudo influenza symptoms PCR + 30WA
P5	35 years old, Civil partnership, no children In an apartment, with no outside premises	10 weeks post partum	Fever, cough, dyspnoea, anosmia, ageusia Screening not done = COVID-19-like symptoms
P6	34 years old, With a partner, no children In a house with a garden	11 weeks post partum	Pseudo-influenza syndrome, anosmia, ageusia PCR COVID-19 +
P7	28 years old Married, no children Apartment with balcony and garden	Pregnant	Anosmia, ageusia (20 WA) No PCR, no serology = COVID-19-like symptoms
P8	31 years old Single, 3 children Social accommodation	4 weeks post partum	Apyrexia, asthenia, myalgia PCR + 30WA
P9	28 years old Married, no children In an apartment, no garden	7 weeks post partum	Rhinitis alone PCR + 28 WA
P10	42 years old With a partner, no children In an apartment, outside yard	1 week post partum	Chest pain, fever, tachycardia Transported by ambulance to the maternity unit PCR + 25 WA
P11	34 years old Married, 1 child Apartment, outside yard	8 weeks post partum	Pharyngitis alone PCR + 28 WA
P12	35 years old With a partner, 1 child Apartment, no outside premises	5 weeks post partum	Rhinitis, dyspnoea PCR + 25 WA

Six themes were identified:

- COVID-19: Minor symptoms, but questioning about an unknown virus
- Identifying oneself as a mother in an anxiety-provoking climate with a loss of social references
- Medical care: fine line between support and ill treatment
- Individual and social resources in the face of adversity
- The central role of the partners
- The unexpected benefits of the lockdown

### COVID-19: Minor symptoms, but questioning about an unknown virus

Most participants reported their COVID-19 symptoms as moderate or “non-serious”. They showed relative serenity regarding the course of the disease. Indifference, even relief at the diagnosis, were also mentioned. *“I thought: OK, well… in fact it was* COVID-19*. What a relief, this is how most people have experienced it: like a bad bout of flu.», P6.*

Worries often reported concerned the health of their unborn child, with the fear of infecting the child, during birth or breastfeeding. *“For me, I was more worried about the baby […] I was worried because I thought I was going to give birth and not be able to breastfeed my daughter», P8.* This fear of passing on the virus also concerned relatives (partner, family). Certain mothers were worried about not being able to look after their other children.

The women seemed to have suffered from a feeling of lack of knowledge about this new virus. Many remained wary about the responses given by health professionals or mass media, or found on the internet, and judged the information unreliable. Although they reckoned that scientific knowledge required constant updates, they experienced negatively the fact that experts and media provided contradictory results over time. *“It’s true that there’s a lot of uncertainty, so it’s never very pleasant, especially when you’re used to controlling everything, being in charge, so er… no it’s not nice.”. P2.*

### Identifying oneself as a mother in an anxiety-provoking climate with a loss of social references

The participants said they had to put to one side what they had imagined they would experience during their pregnancy. Giving up daily activities was complicated to manage. Certain participants who wished to continue working during their pregnancy found it hard to cope with the cessation of work imposed by the crisis, their illness, or the fact of being pregnant. A clear decrease in physical activity among women practicing sport was also difficult, particularly because of the physical consequences. They felt isolated during their pregnancy. Restrictions on movement were experienced negatively, with a feeling of forced confinement, particularly with the presence of other children in the household and the absence of child-minding facilities. *“It’s difficult to manage. Even if the apartment is large, we still have the feeling that we’re going round in circles and it generates anxiety with no particular reason, but the fact of going round in circles is just stressful.», P11.*

During lockdown, the participants found it hard not to be able to see their relatives. They often mentioned the absence of their mother. *“The absence of my family was really hard, because in fact they didn’t really see me pregnant», P10.*

Women feared a brutal break with the world they knew before the pandemic. They reported a “change in atmosphere” and an anxiety-provoking loss of social references.

*“You also have to renounce the fantasy that you build up when you’re going to have a baby. I mean, you think that you will be able to do loads of things, see lots of people, well, just be able to live a little.” P5* ***“*** *Not only did we change, our life changed, everything was changing around us […] So, there it was, nothing was left… becoming a mother in a world where… there was nothing left! It’s… it wasn’t the same world any more.” P5.*

### Medical care: fine line between benevolent support and ill treatment

Maternity departments seemed to have played a major role in supporting women: they were the first resort and the main source of reassurance. Participants appreciated sharing of information by professionals and the availability of the staff. They appreciated particularly telephone follow up sessions, and were disappointed and felt abandoned when hospitals failed to provide it. *“I had direct contact with the doctors. It was really nice. Every day, I was able to talk to a health professional. (..) I found it really reassuring, and I can’t see what they could have done better to take things in hand.” P2.*

The fear of “disturbing” the medical staff, already overwhelmed, was reported several times. Participants were disappointed when prenatal clinics were replaced by telemedicine, which they felt was not reassuring.

Changes in practices, such as wearing a face-mask during labor and pushing efforts, were generally seen as problematic. Regarding the mask: *“It’s true that to catch your breath, it wasn’t easy, even if I had to push only three times, I had the feeling that I was lacking air when I tried to get my breath back”. P9.* A major concern expressed by participants was the risk that their partner might not be allowed to attend the birth, which was almost unanimously felt as intolerable. All these measures were difficult to accept by couples, and sometimes they did not understand the rationale behind these pragmatic decisions. The lexical field related to catastrophe, nightmare, death and trauma was prominent to talk about birth and post-partum hospitalization in the context of the pandemic. *“I felt that it was like coming out of a nightmare, because it was quite traumatic, physically and of course mentally as well (…). I was in tears because my husband wasn’t with me and I was afraid and I couldn’t take it any more. I felt as though my heart was going to stop; it was too much.” P10.*

This revealed a major side effect of safety measures implemented in hospitals to prevent the spread of COVID-19, which, according to participants, bordered on medical violence. *«The bad side of Covid, it was really down to the fact that they completely ruined the birth of my baby, with not good reason whatsoever.” P4.*

### Individual and social resources in the face of adversity

The women interviewed resorted to several strategies to cope with COVID-19. Those who did not feel concerned about the risk of a “severe form” felt protected and did not feel as vulnerable as the women they identified as frightened with a severe form. Others put things into perspective depending on their previous history of childbearing. Primiparous women pointed out that it was simpler not to have other children to look after, and multiparous women stated they were lucky to have already experienced pregnancy so that they were not in unknown territory. Others gave precedence to their maternal role over their role as women, considering that if their baby was well, there was no reason to complain.”*For me the most important thing is that my baby is well, and from there on, OK.” P2.*

Certain women had already experienced serious illness in their lives, which enabled them to put things into perspective or to feel better prepared. Many women declared felt relieved after having COVID-19. It was no longer an abstract threat but a condition they experienced and overcame. They were reassured at the thought of being protected via lasting immunity.

Housing conditions seemed to have a substantial role in the experience of lockdown. Women belonging to higher socio-professional categories felt privileged. *“On top of everything, the weather was good. (…) I had a deckchair under my cherry tree, able to eat my cherries, in peace. A garden makes all the difference.” P6.*

Participants declared they adapted to lockdown by using digital technology to maintain social contacts. Digital means served to communicate on changes in the pregnant person’s body via photos and videos and helped maintaining contact to with friends and family by sharing news on the ongoing pregnancy, the birth and the first moments spent with the baby. *“We made videos, but it was totally different in fact.” P7.*

### The central role of the partner

The partners, who were often mentioned, seemed to have played an important supporting role during these difficult times for a great majority of women. *“My partner, who is often absent, he was always here with me, we were together, and for the pregnancy it was really great. He was really super present with the baby in my belly, in fact.», P10.*

Other women felt their partner failed to give support, and they expressed a feeling of solitude and resentment. *“Basically, in fact, we’ve never been as close physically, but despite that, I have never felt that lonely before», P11.*

The fact that partners were denied the right to be present at prenatal clinics, ultrasound examinations, the post-partum ward, the operative theatre in case of a caesarean, and even the birth room was experienced as an injustice, depriving fathers of their legitimate involvement in the pregnancy and birth process. “*It was really hard for him. In fact, those moments, small as they may be, were taken away from him. It’s difficult, because for us, it was a shared project, we really experience things in life together… and I felt I was being more privileged than him”. P7.*

This feeling was reinforced by the fact that these measures did not seem to be evidence based. Hearsay around the prohibition of the fathers’ presence at birth was the cause of further preoccupation, anticipatory anxiety, even if in the end they were allowed to attend the birth.

Contrastingly, the fact that anti COVID measures kept the partner at home in the post-partum period was good news to participants. They welcomed this kind of a forced parental leave, and declared it helped forming family ties and putting parents on an equal man-woman footage after the birth of the baby. *“I think that in the end, it allowed us to be on an equal footing from the start. Because he saw that I didn’t know anymore than he did why she, [the baby] was crying, we were trying different things… he was also able to calm her down.” P9.*

### The unexpected benefits of the lockdown

Despite all this inconvenience, many participants considered lockdown as an opportunity. On the one hand, during pregnancy, they appreciated to have the opportunity to stay at home with their family. In the course of pregnancy, lockdown enabled expectant mothers to take time for themselves and prepare for the arrival of their baby. They enjoyed being able to focus on their couple and family. *“It did me a lot of good to be able to put my feet up a bit, to make the most of things, to pay more attention to my pregnancy, so that was good.” P7.*

On the other hand, social distancing enabled them to get rid of traditional social constraints in the early post-partum period. The prohibition of post-partum visits from the wider family and friends was received with ambivalence. Many participants resented the prohibition of postpartum visiting, resigning themselves to being deprived of the attentions triggered by having a baby. At the same time, the prohibition of physical contacts was felt to be beneficial in nearly all cases. The post-partum period was described as quieter, less tiring and more respectful of privacy than expected. *“There were fewer visits in fact. Er… well visits can be very tiring, so for me, I was quite happy for the visits to be banned […]. When you’re tired, when you’re… when you’re aching all over, you don’t necessarily want to see any visitors, right?” P10.* This feeling was reinforced by the fact that, very often, the post partum period was harder than what they had expected. Lockdown was a “good excuse” to stay quietly at home. Parents seized this opportunity to concentrate on the newborn baby, to protect it in their own way, without having to justify themselves to their entourage and to society. They were able to discover their child at their own pace, and build a privileged relationship. *“It’s not a bad thing to start finding our marks, just the three of us, so that later we can integrate the other family members. Being able to have a quiet time the three of us in fact.” P7.*

Others verbatim are available in Table 2.

**Table 2:** Boxes of verbatim

**Box 1**	***-COVID 19: Minor or moderate symptoms, but questioning about an unknown virus***
Anxiety related to the health of the child to be born and relatives	*“For me, the first worry that I had when I left for A&E, was to find out whether he was ok (the baby)”. P1* *“I was afraid that my husband might die, you know. When I left with the ambulance, I thought to myself, maybe this is the last time I’ll ever see them. Either because I was going to die, or because they were going to die… I could see death everywhere, it was horrible”. P10*
Fairly unreliable, even contradictory information	*“Because it was the same thing for babies on the news, they were saying: blablabla.. fœtuses are not at risk, blablabla, and then, two weeks later we heard there had been one case.” P1*
**Box2**	***-Identifying oneself as a mother in an anxiety-provoking climate with a loss of social references***
Giving up on daily activities	*“But it’s true that having to stop work, and then stop sport on top of having to stay at home, it was complicated.”P1*
Isolation, restriction in freedom, missing the family	*“Frankly I found it…it was hell being at home on my own.” P4* *“So, I felt lonely on my own during my pregnancy because of this. I missed my mother particularly; I missed her a lot.” P10*
Giving up the pregnancy that was planned in a world before COVID-19	*“There wasn’t the fun side of having a first baby, going to look at things… I don’t know. Going to look at buggies, perhaps… We did everything on the Internet… That was it.” P2* *“ I would have liked to have had the sensation of what it is like to float at the deep end of the pool with a big belly, when your feet can’t touch the bottom”. P2*
Break with the world before, loss of references	*“ I was worried about the world and globalisation, about everything surrounding us. Being pregnant in such times, it was really horrible.” P10*
**Box 3**	***-Medical care: fine line between indispensable support and ill treatment***
Tele-consultation: not satisfactory	*“I didn’t particularly appreciate the follow-up on the phone, I must say. It would have reassured me if I had been examined a bit for my baby. On the phone, it’s not at all the same thing.” P2*
Fear of disturbing	*“No, in fact, so long as I didn’t have a temperature, I didn’t dare, I didn’t want to kick up a fuss.», P7*
Ill treatment, obstetrical violence during birth	*“Oh well, that was a catastrophe, it was a total catastrophe. The anesthesiologist was screaming at me because I was not obeying him.” P4*
**Box 4**	***-Individual and social resources in the face of adversity***
Distancing	*“It’s true that the* COVID-19 *pandemic… we were rather serene about it… We are not in the risk-prone categories and I still think that children are not part of the risk-prone categories.” P9*
Previous history of serious illness	“*I have seen worse! I have a very loaded medical history, so I’ve seen worse.” P1*
COVID-19 infection	*“I was happy to have had it because I thought: Ok, so that’s done! We’ve all had it. We are… in a way, well, safe now.” P10*
Digital social links insufficient	*“I also missed having that kind of contact… even if we could use the phone, it’s not the same.” P11*
**Box 5**	***-The central role of the partners***
Supporting role	*“Being with someone, it was reassuring. I felt protected.” P2*
Not being allowed to take part in the pregnancy follow-up experienced as an injustice	*“I’m not sorry for myself, I’m sorry for him. These are moments you can’t relive afterwards…” P2* *“We live together, we lived through lockdown together, so we didn’t understand these things, why should we by separated for this medical follow-up?” P7*
Intense anxiety at the idea of not being allowed to attend the birth	*“We weren’t sure that the father would be able to attend the birth, and it’s something that I would have felt I was robbed of, this particular moment. Even the first days after my daughter was born. It’s for him, really, I would have been sorry to be with her and not him…», P9*
**Box 6**	***-The unexpected benefits of the suspension of social norms***
Spending time as a couple or as a family	*“Being able to do things once more together, without the pressure of having to go out, of absolutely having to do something. For our part, we loved lockdown.” P9*
A cocoon and a privileged relationship with the new-born baby	*“An enriching (experience) in the sense that I really experienced the end of the pregnancy cut off from the rest of the world, and the birth, and my daughter, without any outside pressure whatsoever. I could discover my daughter, without the outside world looking on, it was great.” P6*

## Discussion

The main worry for women affected by COVID-19 in our study was not the risk the infection carried to their own health, but the fact that they could transmit the disease, especially to their relatives, and above all, the general disruption resulting from the pandemic and the lockdown. Our results were similar to those of Corbett, concerning the serious worries of future mothers during the pandemic on the subject of their family’s health (including the child to come), and on changes imposed on lifestyle (social isolation, work from home, commuting difficulties and child-minding) [14]. The distress of women coping with isolation could be explained by the changes in the process of identity construction. which is achieved in part by how other people view it, via different “*pregnancy markers*” [15]. For instance, receiving attention or preparing for the baby’s arrival are phenomena that place pregnancy in the sphere of a woman’s social standing and give substance to the imminent birth of the baby. In order to feel “pregnant”, women need to be seen [16]. This was observed in the present study, via the need to maintain a visual link with others during lockdown (photos and videos shared showing the changes in the body during pregnancy).

In our study, participants’ utmost concern was the idea of being separated from their partner at crucial moments including prenatal visits, ultrasounds examinations, birth, and the postpartum. Some of our findings are in accordance with previous papers, including the fear of pregnant persons to be separated from the partner and other children, the difficulty of coping with limited social interactions, the demand for support from health institutions. These feelings were expressed by COVID-19 positive [17] and by COVID-19 negative pregnant persons [4, 7, 14, 18, 19], in different settings including Italy [17], Ireland [14], Turkey [4, 5], Australia [18, 19].

The traumatic experience of pregnant women recruited in our study was related to the unexpected side effects of the preventive measures implemented to limit viral transmission, with a lack of information about these measures. Women were asking themselves whether these measures were evidence-based, or resulted from an unscientific precautionary principle or even from an authoritarian and arbitrary decision. This, in our opinion raises the question of medical and institutional violence. Becoming a mother during the COVID-19 pandemic amounted to facing of adversity by calling upon various resources. Our results underline the importance of gynaecology-obstetrics units as a “monitoring institution” for pregnant women [15]. The women’s relationship to medicalization was ambivalent. Whereas women tended to be apprehensive of excessive medicalization during a « normal” pregnancy, medicalization was welcome concerning COVID-19, provided professionals were available, empathetic, and willing to share reliable information.

Our results were in accordance with the evolution of parental roles in society. In 1988 [20], a study from IPSOS, a French consulting firm, reported that 71% of pregnant women interviewed did not wish for their husband’s presence “at all costs” during birth. Times have changed [21]. In our study, women reported that the exclusion of fathers from the pregnancy follow-up was experienced negatively by both partners. This is in accordance with other studies, in which future fathers reckoned that attending prenatal ultrasound, was paramount for constructing parenthood via the tangible apprehension of the child [22]. In our study, the thought that fathers could not take part in the birth was almost unanimously felt as intolerable. Although they had been affected by COVID-19, women felt more privileged than their partners, and resented the injustice of their exclusion from the pregnancy and childbirth process. We could discuss here about “paternal commitment” [23]. This term referred to the current trend for fathers to be more involved in the domestic space, and in particular in caring for the children. It would seem that they now, more than in the past, are expected to be true actors in parenthood. Our main themes are in accordance with a recent qualitative metasynthesis with mostly American articles, describing the negative experience of women during the pandemic [24].

Our study, centered on women who had COVID-19, had two unexpected findings. First, participants considered work from home was a blessing: it extended the duration of the maternity leave, amounted to a de facto paternity leave, which facilitated gender equality in household chores and baby care. Second, the pandemic social distancing helped participants escaping from behavioural and social constraints, including the unspoken rule that they should welcome greetings from friends and family, despite being exhausted by their recent birth. Parenting is underpinned by an intimate and personal dimension which intertwines with the public and socially normed dimension [25]. “Parenting skills” refer to attitudes and behaviors that society expects from a “good parent” [26]. The family sphere (at large) is the main source of judgment and injunctions made to new parents [26]. Lockdown might have protected people against social constraints in general. The declarations of the young mothers we interviewed suggest this applied to the expected parental behavior in front of friends, family, or neighbors. These unexpected results might reveal the social pressure put to new parents.

### Strengths and limitations

The interviews were individual, long and fruitful, which enabled the most delicate aspects of the experience to be discussed. The course of the interviews was open, which helped the women to express themselves and limited the influence of the researchers during the interviews. The triangulation, necessary for the scientific validity of the approach, was achieved on two levels: data collection (4 different researchers carried out the interviews) and analysis (3 researchers conducted the analyses individually and then pooled them). All data collection and analysis was discussed by the research team. To our knowledge, no qualitative study on SARS-CoV-2 infection during pregnancy has been carried out in France. An IPA requires about ten interviews, provided they are sufficiently long and enable in-depth access to the participants’ experiences. Twelve interviews were achieved and sufficiency was obtained on the present research theme. There is no bias in qualitative analysis, since the research is not meant to be objective. However, it could be pointed out that the participants in this study had by definition “agreed to take part”. This could suggest that they judged their experience interesting, enriching or traumatic. Also, we did not study the experiences of women having gone through a severe form of COVID-19.

## Conclusions

The COVID-19 pandemic has been an unprecedented phenomenon as a result of the high contagiousness of SARS-CoV-2 and the generalized lockdown it caused. This study, the only one performed in France that the authors are aware of, may have provided keys for adapted and empathic medical care for pregnant women in times of health crises. Interviewing partners of pregnant women affected by COVID-19 should provide us a direct access to their thoughts and difficulties in parenthood process during pandemic, in heterosexual or non -heterosexual relationships. Furthermore, it contributes to outlining the contours of parenthood today. Qualitative metasynthesis using qualitative researches performed all over the world could be an asset to understand better what pregnant women and their companions lived in this period of SARS-CoV-2 pandemic, to support them the best, facing both the virus and a new parenthood in this world of uncertainty.

## Data Availability

The datasets used and/or analysed during the current study are available from the corresponding author on reasonable request.
